# Molecular Phylogeny and Biogeography of *Percocypris* (Cyprinidae, Teleostei)

**DOI:** 10.1371/journal.pone.0061827

**Published:** 2013-06-04

**Authors:** Mo Wang, Jun-Xing Yang, Xiao-Yong Chen

**Affiliations:** 1 State Key Laboratory of Genetic Resources and Evolution, Kunming Institute of Zoology, Chinese Academy of Sciences, Kunming, Yunnan, China; 2 University of Chinese Academy of Sciences, Beijing, China; University of Basel, Switzerland

## Abstract

Fierce predatory freshwater fishes, the species of *Percocypris* (Cyprinidae, Teleostei) inhabit large rivers or lakes, and have a specific distribution pattern. Only a single species or subspecies occurs in each large-scale drainage basin of the Southeastern Tibetan Plateau. In this study, the molecular phylogenetic relationships for all but one of the described subspecies/species of *Percocypris* were investigated based on three mitochondrial genes (16S; COI; Cyt *b*) and one nuclear marker (Rag2). The results of Maximum Likelihood and Bayesian Inference analyses show that *Percocypris* is a strongly supported monophyletic group and that it is the sister group of *Schizothorax*. Combined with analyses of morphological characters, our results suggest that *Percocypris* needs to be reclassified, and we propose that six species be recognized, with corresponding distributions in five main drainages (including one lake). In addition, based on the results of the estimation of divergence times and ancestral drainages, we hypothesize that *Percocypris* likely originated in the early Miocene from a paleo-connected drainage system containing the contemporary main drainages of the Southeastern Tibetan Plateau. This study suggests that vicariance (due to the uplift of the Tibetan Plateau modifying the large-scale morphologies of drainage basins in the Southeastern Tibetan Plateau) has played an important role in the speciation of the genus. Furthermore, external morphological characters (such as the length of the fins) and an internal trait (the position of pterygiophore) appear to be correlated with different habitats in rivers and the lake.

## Introduction

The species of *Percocypris* (Cyprinidae, Teleostei) are fierce predatory freshwater fishes inhabiting large rivers or lakes, in southwestern China and northern Vietnam. Members of the genus have a specific distribution pattern, that is, there is only one species or subspecies in each drainage as follows (Chinese names in brackets): Upper Yangtze River (Jinsha Jiang), Mekong River (Lancang Jiang), Salween River (Nu Jiang), Upper Pearl River (Nanpan Jiang), Red River (Yuan Jiang). The genus thus appears to be an ideal system to study how historical geologic or geographic events of the relevant drainages including the famous Three Parallel Rivers of Yunnan Protected Areas (Salween, Mekong and Upper Yangtze rivers) influenced the biogeography of freshwater fishes.

However, even the basic taxonomy of how many species of *Percocypris* exist has not been resolved. Chu [1] erected *Percocypris* for *Leptobarbus pingi* Tchang (1930) [2]. In the same year, Tchang [3] described *Barbus regani* (subsequently treated as *P. pingi regani*; [4]–[7]) from Fuxian Lake. Cui & Chu [6] described *P. pingi retrodorslis* from Mekong and Salween rivers, and presented a classification system of one species with three subspecies that was adopted by other Chinese researchers (e.g., [7]). Nevertheless, Kottelat [8] regarded the three subspecies as three species with the scientific names of *P. pingi*, *P. regani* and *P. tchangi* (*P. pingi retrodorslis* treated as a synonym of *P. tchang*), and pointed out that the species *P. tchangi* Pellegrin & Chevey 1936 [9] (described from Red River) was apparently overlooked. The basic disagreement over the classification of *Percocypris* – of whether it consists of one species with three subspecies (*P. pingi pingi*, *P. pingi regani* and *P. pingi retrodorslis*; [6]) or three species (*P. pingi*, *P. regani* and *P. tchangi*; [8]) – needs to be resolved. In this study, we provisionally follow the classification system of Cui & Chu [6], that is, *P. pingi pingi* (Upper Yangtze River), *P. pingi regani* (Fuxian Lake, Upper Pearl River) and *P. pingi retrodorslis* (Mekong and Salween rivers).

The studies cited above on the taxonomy of *Percocypris* relied entirely on morphological characters. However, molecular studies on *Percocypris* to date have utilized only collections of *P. pingi pingi* from one locality (Hejiang, Sichuan Prov.) and *P. pingi retrodorslis* from one locality (Baoshan, Yunnan Prov.). The sample of *P. pingi pingi* was used in the molecular phylogenetic analyses of Wang et al. [10], Kong et al. [11] and Li et al. [12], which were based on the Rag2 [recombinant activation gene 2], *S6K1* [ribosomal protein S6 kinase 1] and 16S [16S ribosomal small subunit] genes, respectively. In addition to *P. pingi pingi*, one sample of *P. pingi retrodorslis* (IHBCY0505008; Baoshan, Yunnan Prov.) was also included in the study of Li et al. [12]. The results of all the three studies suggested that *Schizothorax* may be the sister group of *Percocypris*. In addition, the monophyly of *Percocypris* was not firmly established by Li et al. [12], due to the particularly weak nodal supports of the clade of *P. pingi pingi* and *P. pingi retrodorslis* (Maximum parsimony bootstrap values  = 56; Bayesian posterior probability  = 0.80/0.82). These studies suffered from incomplete taxon sampling, with only two samples, at most, included. Moreover, only a single gene was used in these analyses; no combined molecular data set was compiled. Thus, there are ambiguities regarding the relationships within this genus, and the monophyly of *Percocypris* has not been convincingly demonstrated.

The potential impact of paleo-drainage basin morphologies on biogeographic patterns of the Tibetan Plateau and East Himalayas has been attracting increasing attention in recent studies (e.g., [13]–[18]). Although *Percocypris* is likely an ideal system for testing the biogeographical hypotheses, the evolutionary history and even the classification of this group were not well understood because of the difficulty of collecting relevant specimens. To the best of our knowledge, there have been few, if any, studies using a fish genus with the particular distribution pattern found in *Percocypris* to investigate biogeographic issues of the Southeastern Tibetan Plateau.

In this paper, a molecular phylogeny was reconstructed including all but one of the putative species of *Percocypris*, based on a combined dataset of three mitochondrial DNA genes and one nuclear gene. The divergence times and ancestral drainage of this group were also inferred using the Bayesian relaxed molecular clock and primary Brooks Parsimony Analysis (BPA) methods, respectively. The objective of this study was to clarify the relationships within *Percocypris* based on the results of multiple molecular phylogenetic methods and the analysis of morphological traits. Furthermore, we attempted to analyze the potential relationship between the evolutionary history of the genus and the change of paleo-drainages surrounding the Southeastern Tibetan Plateau. On the basis of the results of molecular and morphological analyses, we suggest that the species of *Percocypris* should be reassigned to six species, the distributions of which coincide with five river drainages and one lake basin (connective with one of the five river drainages). In addition, our results suggest that the genus originated during a time when the present-day drainages inhabited by *Percocypris* were connected to each other. We hypothesize that the changes in the large-scale morphologies of paleo-drainages basins of the Southeastern Tibetan Plateau played a significant role in the speciation of *Percocypris*.

## Materials and Methods

### Ethics statement

All the animal samples were obtained in compliance with “Law of People's Republic of China on the Protection of Wildlife” and “Regulations for the Implementation of the People's Republic of China on the Protection of Aquatic Wildlife”. The samples were processed with the approval of the ethics committee of the Institutional Review Board of Kunming Institute of Zoology, Chinese Academy of Sciences. All specimens are stored in Kunming Institute of Zoology, Chinese Academy of Sciences (KIZ) and were used with the permission of KIZ.

### Taxon sampling

**Figure 1:**
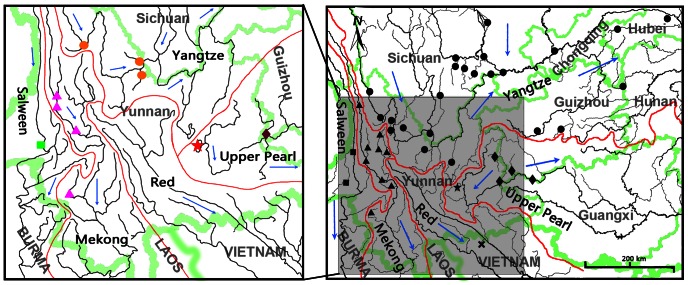
Distributions of *Percocypris* and molecular samples. Red lines indicate major river basins; blue arrows denote general direction of water flow; green lines indicate boundaries. (Right map) Extant distributions of *Percocypris* based on literature records and localities of specimens: dots – Upper Yangtze River, five-pointed star – Fuxian Lake, diamonds – Upper Pearl River, triangles – Mekong River, squares – Salween River, cross – Red River; (Left map) Geographic distribution of molecular samples of *Percocypris* used in this study: orange dots – Upper Yangtze River, red five-pointed star – Fuxian Lake, purple diamond – Upper Pearl River, pink triangles – Mekong River, green square – Salween River.

Thirty eight individuals of *Percocypris* were measured in the morphometric study and forty three specimens of *Percocypris* were examined for the osteological features. Thirty four specimens of *Percocypris* were used in the molecular analysis, representing three subspecies of the nominal *P. pingi* from Fuxian Lake, Upper Pearl River, Upper Yangtze River, Mekong River and Salween River (the collection sites are shown in Figure 1). The specimens were collected by electro-fishing and/or seining mainly from 2003 to 2011, and were subsequently deposited in KIZ. Although many localities including the type locality (the city of Laocai in Vietnam) of Red River basin were sampled numerous times at different seasons every year, we failed to acquire any specimens of *P. tchangi*. Following recent studies involving *Percocypris* [10]–[12], *Schizothorax meridionalis*, *S. waltoni*, *Onychostoma simum*, *Spinibarbus denticulatus*, *Barbonymus schwanenfeldii*, *Sinocyclocheilus tingi*, *Cyprinus pellegrini*, *Carassius auratus*, *Tor douronensis*, *Labeo stoliczkae* and *Danio rerio* were included as outgroup taxa. All of the ingroup members (except *P. tchangi*) and three outgroup taxa (*Spinibarbus denticulatus*, *Cyprinus pellegrini*, *Labeo stoliczkae*) were sequenced in this study, and the sequences deposited in GenBank (Listed in Table S1 along with sequences of other outgroup taxa obtained from GenBank).

### Morphological analyses

In order to explore the morphological variation among the different species and the various habitat types of *Percocypris*, the morphological analyses included both external morphological measurements and aspects of the skeletal system.

**Figure 2:**
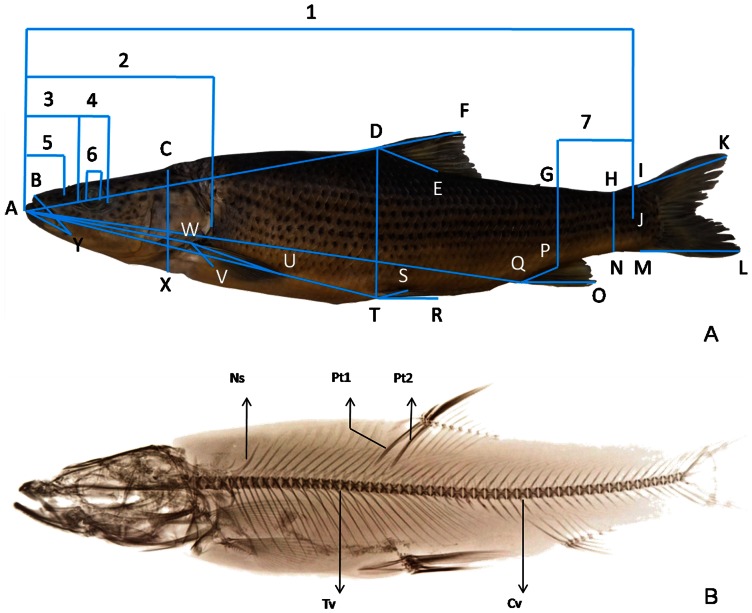
External morphological measurements and internal skeletal traits. (A) External morphological measurements used for morphometric analysis in this study: 1, standard length (SL); 2, head length (HL); 3, snout length (SNL); 4, eye diameter (ED); 5, prenaris length (IPNW); 6, eye-ball diameter (EBD); 7, caudal peduncle length (CPL); AD, predorsal length (PL); DE, dorsal-fin base length (DBL); DF, dorsal fin length (DFL); AW, prepectoral length (PPTL); WV, pectoral-fin base length (PTBL); WU, pectoral fin length (PTFL); AT, prepelvic length (PPVL); TS, pelvic-fin base length (PVBL); TR, pelvic fin length (PVFL); AQ, preanal length (PAL); QP, anal-fin base length (ABL); QO, anal fin length (AFL); HN, caudal peduncle depth (CPD); IK, upper lobe of caudal fin length (UICL); ML, lower lobe of caudal fin length (LLCL); CX, head depth (HD); BY, upper jaw length (UJL); AY, lower jaw length (LJL); the other measurements followed Chu & Cui [5] and Zhao & Zhang [19]: body depth(BD); caudal peduncle depth at the terminal of Anal fin base (CPDTA); middle caudal fin length (MCL); head width (HW); interorbital width (IOW); width between posterior naris (IPONW); mouth width (MW); maxilla barbel length (MBL); rictal barbel length (RBL). (B) Internal skeletal traits analyzed in this study: Ns, neural spine; Tv, trunk vertebrae; Cv, caudal vertebrae; Pt1, 1^st^ dorsal pterygiophore; Pt2, 2^nd^ dorsal pterygiophore.

For the external morphological analysis, 38 individuals of *Percocypris* were measured for 34 morphological variables. These were recorded to the nearest 0.1 mm using digital calipers following the methods of Chu & Cui [5] and Zhao & Zhang [19]. The 34 morphological measurements are shown in Figure 2. Summary statistics for all the morphological characters were calculated with the statistical program SPSS 17.0 (SPSS for Windows, Chicago, IL, USA) for the Principal Component Analysis (PCA) after scaling according to standard length.

As a significant diagnostic morphological characteristic for species/subspecies of *Percocypris*, skeletal system images of 43 specimens of *Percocypris* were obtained and the osteological features (Figure 2) were observed and counted on a radiograph (X-ray film) taken by molybdenum target radiography.

### Molecular methods

Fin tissue samples were frozen in 95% ethanol at −80°C until used. Total genomic DNA was extracted from the alcohol-preserved tissue with the proteinase K digestion and sodium dodecyl sulfate (SDS) extraction, high salt or phenol isolation and isopropanol precipitation procedure [20]. Three mitochondrial genes (16S, COI [cytochrome oxidase gene subunit I] and the complete Cyt *b* [cytochrome *b*],) and one nuclear protein-coding gene (Rag2) were amplified using polymerase chain reaction (PCR) with primer sequences given in Table S2.

All the mitochondrial and nuclear DNA PCR-amplifications, performed in 50 μl volume (37 μl of double distilled water, 5 μl of 10× PCR reaction buffer, 3 μl of 2.5mM dNTPs, 2 μl of BSA [bovine serum albumin], 1 μ of a 10 μM solution of each primer, 2.0 U *Taq* DNA polymerase [Sangon Inc., Shanghai, China] and about 100 ng of DNA template), were carried out using the following procedures: an initial denaturing step of 5 min at 94°C, followed by 35 cycles with denaturing 30 s at 94°C, annealing 60 s at 55°C, 50°C, 50°C and 55°C (for 16S, COI, Cyt *b* and Rag2, respectively), extending 60 s at 72°C and a final extension step of 10 min conducted at 72°C. After electrophoresis through a 1.5% agarose gel, all amplified DNA fragments were purified using UNIQ-10 spin column DNA gel extraction kit (Sangon Inc., Shanghai, China) according to manufacturers' instructions. Using the corresponding primers (Table S2), each fragment was sequenced in both directions with the BigDye Terminator Cycle Sequencing Kit (Applied Biosystems) on an ABI 3730 automated sequencer.

### Sequence alignment, data partitioning and model selection

Sequences of all genes were proofread and assembled with the DNA analysis package DNASTAR Lasergene Seqman and EditSeq version 7.1 (DNAStar Inc., Madison, WI). Alignment of protein-coding sequences (COI, Cyt *b* and Rag2) was conducted using Clustal X 1.83 [21] with default settings, after which the DNA sequences were translated to amino acids residues with the software MEGA 5.0 [22] to test for the absence of premature stop codons or indels, and subsequently checked by eye to maximize positional homology. For 16S ribosomal genes, the alignment was initially performed using the program MUSCLE [23] with default parameters and also further revised by eye. All sequences obtained in this study were deposited in GenBank database (Table S1 for accession numbers). For each fragment, after alignment, basic compositional information was estimated with the software MEGA 5.0 [22].

We partitioned the dataset into two, three, four, five, six, seven or ten partitions based on multiple partitioning strategies (see Table 1 for partition identities). The best fitting evolutionary model of each partition in all data partitioning strategies using Bayesian information criteria (BIC; [24]) was determined with the software jModeltest version 2.1.2 [25] (selection of the 88 candidate substitution models) and Kakusan4 [26] (selection of the 56 candidate substitution models). BIC was chosen to select a model because of its high accuracy and precision [27] and its tendency to select simpler models than AIC [28]–[30]. We also compared the nonpartitioned, proportional and separate models [31] on each partition using Treefinder [32] in Kakusan4 [26].

**Table 1:** List of partitioning strategies used in the partitioned Bayesian analyses.

#	Partition strategy	Partition identity
P_2_	By mitochondrial and nuclear gene	16S+COI+Cyt *b*; Rag2
P_3_	By non-coding mitochondrial, protein-coding mitochondrial and nuclear gene	16S; COI+Cyt *b*; Rag2
P_4_	By gene	16S; COI; Cyt *b*; Rag2
P_5_	Based on the proposed method of Li et al. [98]	16S; COI_12+Cyt *b*-12; COI_3+Cyt *b*_3; Rag2_12; Rag2_3
P_6_	Based on the analysis of our combined dataset using PartitionFinder [99]	16S+Cyt *b*_1; COI_1; COI_2+Cyt *b*_2; COI_3+Cyt *b*_3; Rag2_12; Rag2_3
P_7_	By gene and separating codon positions 1 & 2 and codon position 3 of protein-coding gene	16S; COI_12; COI_3; Cyt *b*_12; Cyt *b*_3; Rag2_12; Rag2_3
P_10_	By gene and codon position of protein-coding gene	16S; COI_1; COI_2; COI_3; Cyt *b*_1; Cyt *b*_2; Cyt *b*_3; Rag2_1; Rag2_2; Rag2_3

The numerical subscripts next to the capital P mean the number of data partitions. The number after COI, Cyt *b* and Rag2 (1 2 3) mean the first, second and third codon position, respectively.

### Phylogenetic analyses

We performed phylogenetic analyses using maximum likelihood (ML) and Bayesian inference (BI) methods. Fourteen (seven [partitioning strategies] * two [selected substitution models]) partitioned BI analyses were performed using the settings below. Bayes Factor [33]–[35] was used to choose alternative partitioning strategies and model selections with jModeltest2 [25] and Kakusan4 [26]. We calculated Bayes Factors by computing the marginal likelihood of log-transformed harmonic means for each BI run (estimated in MrBayes using the “sump” command) in Tracer v. 1.5 [36]. The value of 2 ln Bayes factors ≥10 are considered to be very strong evidence supporting the alternative strategy [37]. The same partitioning scheme and evolutionary models chosen by Bayes Factors were used in both ML and BI analyses. Gaps in the 16S dataset were treated as missing data.

BI analyses were conducted using MrBayes v3.2.1 [38], with following settings: two Markov chain Monte Carlo (MCMC) runs of four chains each for 3 million generations, a sampling frequency of 100, and a diagnosing frequency of 1,000. All parameters between partitions except topology and branch lengths were unlinked. The appropriate burn-in fraction and convergence of the MCMC chains were graphically assessed by evaluating the stationary phase of the chains using Tracer v. 1.5 [36] and the web-based program AWTY [39]. The final consensus tree and Bayesian posterior probabilities (PP) were generated with the remaining tree samples after discarding the first 60% of samples as burn-in.

For the ML method, we conducted partitioned analyses with the software GARLI 2.0 [40] using the optimal model of evolution for the five partitions with the models and substitution rates unlinked between partitions. To estimate the best tree, five replicate searches were run with each replicate run for five million generations (stopgen  = “5000000”). 100 nonparametric bootstrap pseudoreplications were performed with the software GARLI and a strict consensus tree was generated from the resulting bootstrap trees with the software PAUP 4.0b 10 [41]. The ML bootstrap probability values (BP) were calculated in PAUP.

The average genetic distances between the clades inferred by phylogenetic analyses were computed by Kimura's two-parameter method [42] with the program MEGA 5.0 [22].

### Divergence time and ancestral drainage estimation

To estimate divergence time within *Percocypris*, the Bayesian relaxed clock method [43] was used in the program BEAST v. 1.7.4 [44]. In order to use the reliable fossils of Cypriniformes, we included an additional 70 taxa for the molecular dating analyses (totally 127 samples; Table S1). *Corydoras rabauti* was used as outgroup. The 16S, COI and Cyt *b* genes were used for our divergence time estimation because these genes have been extensively used in studies of phylogeny in Cypriniformes and have been more widely sampled than the Rag2 gene. We partitioned the dataset into five partitions as we did in the phylogenetic analyses and chose the models selected by BIC for each partition (Table S3) after Kakusan4 analyses. BEAUti v. 1.7.4 [44] was used to generate the input files for the analysis. Analysis was conducted with uncorrelated lognormal relaxed molecular clock model, with partition-specific substitution models, a Yule speciation process for the tree prior, random starting tree but constraining the ingroup to be monophyletic, the prior of mean substitution rate (ucld.mean) fixed to CTMC Rate Reference [45], and with the most recent common ancestor (MRCA) of the four clades associated with fossil calibration points (see below) treated as lognormal distributions.

We chose the oldest and most unambiguous fossil records for constraints of the age of the root node, setting the latest date of the fossil record as minimum and a soft maximum with lognormal distribution: (1) *Barbus bohemicus* and *Barbus sp*. were reported from Czech Republic and dated as 18–19 million years ago (Mya) [46]. Thus, the split between *Barbus* and its sister group (*Luciobarbus* and *Capoeta*; e.g. [47], [48]) was at least 18 Mya and was constrained to a minimum of 18 Mya (log (mean)  =  “0.35” log (stdev)  =  “1.0” offset  = “18.0”). (2) Four fossils of *Mylocheilus inflexus* and five fossils of *Mylocheilus robustus* were recorded from Tortonian (10.5–11.5 Mya; [46]) in U.S.A. Therefore, we assumed that the split between *Mylocheilus* and its sister group (*Pogonichthys*; see Schönhuth et al. [49]) occurred at least 10.5 Mya (log (mean)  =  “0.5” log (stdev)  =  “1.0” offset  = “10.5”). (3) The oldest known *Mylopharyngodon* fossil (*Mylopharyngodon wui*), paleomagnetically dated to 12.5 Mya [50], is known from the middle Miocene of China. *Ctenopharyngodon idella* is the sister group of *Mylopharyngodon piceusi* [51], [52], and we assumed that the MRCA of *Mylopharyngodon* and its sister group was at least 12.5 Mya (log (mean)  =  “0.5” log (stdev)  =  “1.0” offset  = “12.5”). (4) The oldest fossil similar to *Myxocyprinus asiaticus* (*Plesiomyxocyprinus arratiae*) was recorded from the early to middle Eocene in China (37.2–40.4 Mya; [46], [53]). The split between *Myxocyprinus* and its sister group (*Carpiodes*, *Ictiobus*, *Cycleptus*, *Catostomus, Minytrema, Moxostoma* and *Hypenteliums*; e.g. [54]–[56]) was set to be not later than 37.2 Mya (log (mean)  =  “1.1” log (stdev)  =  “1.0” offset  = “37.2”). In addition, a secondary calibration point was used on the root age of the tree. The root age was set using a normal distribution (mean  = “170.5” stdev  = “10.6”) based on the divergence time of Otophysi (the split between ingroup and outgroup [*Corydoras rabauti*]; 170.5 Mya; HPD [Highest Posterior Density] 153.1–187.9) estimated by Near et al. [57].

Two independent MCMC chains with a total of 50 million generations, sampled every 5000 generations were run using the corresponding substitution model for each partition. Convergence to the stationary distribution was assessed by inspection of log output files using Tracer and plots of tree files using AWTY. After discarding the burn-in of 25 million generations, the remaining tree samples of the four converged runs were combined using LogCombiner. The maximum clade credibility tree was calculated using TreeAnnotator and visualized with the software FigTree v.1.3.1 [58].

**Figure 3:**
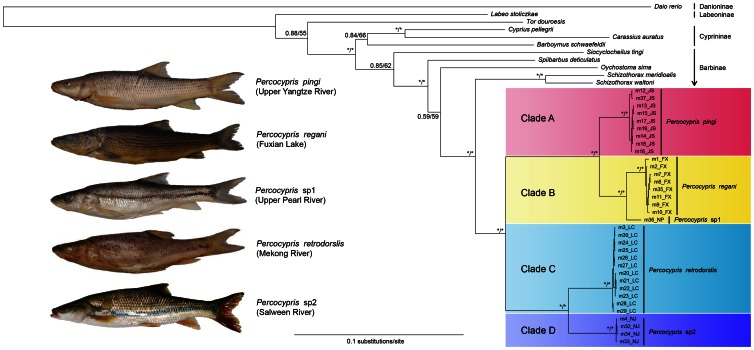
The topology generated by the partitioned Bayesian analysis inferred from the combined data set. The nodal numbers or symbol are Bayesian posterior probability and ML bootstrap values as node supports. The asterisk (*) indicates posterior probability ≧0.95 and ML bootstrap values ≧70%. JS, NP, LC, NJ and FX in the name of sample (e.g. m12_JS) stand for Jishan River, Nanpan River, Lancang River, Nujiang River and Fuxian Lake, respectively.

We used BPA method [59] to estimate the ancestral distribution area and investigate the evolutionary history of *Percocypris*. Based on the present-day drainage distributions of *Percocypris*, five units (Upper Yangtze River, A; Upper Pearl River, B; Fuxian Lake, C; Mekong River, D; and Salween River, E) were defined. The optimized phylogeny (derived from Figure 3) was converted into drainage cladogram. Data matrix to the primary BPA was prepared from the cladogram.

## Results

### Variation in external morphological measurements and internal skeletal system

**Figure 4:**
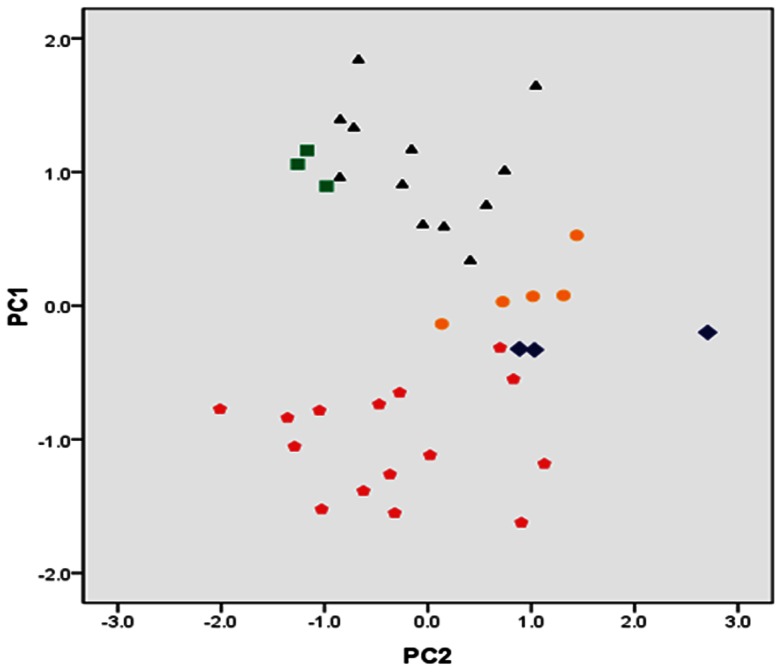
Scatter plot of the first principal component (PC1) vs. the second principal component (PC2). The species from Fuxian Lake (red pentagons), Upper Pearl River (blue diamonds), Upper Yangtze River (orange dots), Mekong River (black triangles) and Salween River (green squares).

The results of the PCA of external morphological measurements are presented in Table 2 and Figure 4. The first three principal components explained a cumulative 75.627% of total observed variance with 56.743%, 12.328% and 6.556% explained by the first principal component (PC1), the second principal component (PC2) and the third principal component (PC3), respectively (Table 2). Furthermore, the length of fins, the distance between the snout and each fin, the length of barbels (maxilla barbel and rictal barbel), the width between posterior nares and the scale of the head (PL, DFL, PPTL, PTFL, PPVL, PVFL, AFL, MCL, UICL, LLCL, HD, HW, IPONW, MBL and RBL; Table 2) contributed most to PC1. As shown in Figure 4, the PCA indicated five distinct clusters corresponding to species from four river basins (Upper Pearl, Upper Yangtze, Mekong and Salween rivers) and Fuxian Lake.

**Table 2:** Variance loadings on the first three principal components (PC1, PC2 and PC3) in the analysis of variation in external morphology for species of *Percocypris* among the main clades.

Characters	PC1	PC2	PC3
BD	0.341	0.341	0.777
CPDTA	0.212	0.373	0.662
PL	**0.833**	−0.268	0.032
DBL	0.275	−0.007	0.244
DFL	**0.786**	0.332	0.064
PPTL	**0.785**	−0.239	−0.299
PTBL	0.63	0.055	0.053
PTFL	**0.873**	0.325	−0.003
PPVL	**0.768**	−0.515	−0.002
PVBL	0.367	0.07	0.335
PVFL	**0.909**	0.249	0.053
PAL	0.473	−0.758	0.253
ABL	0.594	−0.071	−0.108
AFL	**0.884**	0.191	0.07
CPL	0.013	0.176	0.554
CPD	0.65	0.195	0.557
MCL	**0.842**	0.083	−0.159
UICL	**0.825**	0.224	−0.142
LLCL	**0.898**	0.17	−0.161
HL	0.664	−0.301	−0.003
HD	**0.9**	0.131	0.064
HW	**0.813**	0.114	0.087
ED	0.343	−0.119	−0.235
EBD	−0.534	−0.127	−0.126
IOW	0.743	0.306	0.117
IPNW	0.039	−0.311	−0.216
IPONW	**0.764**	0.163	0.118
SNL	0.269	−0.289	0.091
MW	0.626	−0.048	−0.309
UJL	0.742	0.164	−0.196
LJL	0.673	0.105	−0.236
MBL	**0.841**	0.25	−0.236
RBL	**0.885**	0.287	−0.043
Variance (%)	56.743	12.328	6.556
Cumulative (%)	56.743	69.071	75.627

The boldface values represent the variances that have the high contribution to PC1.

The number of neural spines before the first pterygiophore was found to differ among the clades of *Percocypris* after the examination on the X-ray film of skeletal system, as shown in Table S4 (in which the counts of meristic characters of forty five specimens are given as well). An additional two-tailed Pearson's bivariate correlation was performed to examine the relationship between the position of pterygiophore and the external position of dorsal fin using the software SPSS 17.0. A significant positive bivariate relationship (Pearson's correlation  = 0.806) was found.

### Sequence characteristics, data partitioning and tree statistics

Including the sequences of eight outgroup species downloaded from the Genbank (24 mtDNA and 8 nuDNA sequences), a total of 1122 bp (base pairs) of 16S, 847 bp of COI, 1140 bp of Cyt *b* and 1236 bp of Rag2 (entirety 4345 bp) were resolved after alignment. For the three protein-coding genes (COI, Cyt *b* and Rag2), no premature stop codons or indels were observed after translation. In addition, no ambiguously aligned regions were found in 16S sequences.

The mean ln likelihood (ln *L*) and Bayes factor comparisons are presented in Table 3. The best partition_model strategy was the most partitioned scheme separated by gene and codon position, with the model selected by Kakusan4 (P_10__K; Table 3). For the BI and ML analyses, the best-fit substitution models for each partion selected by BIC in Kakusan4 are given in Table S3. The BI runs in MrBayes produced a posterior distribution with ln *L* = −17341.22. The ML analysis generated the most likely tree with ln *L* = −17237.64753.

**Table 3:** Comparisons of alternative partitioning strategies and model selections.

Partition_Model	-ln *L*	P_2__j	P_2__K	P_3__j	P_3__K	P_4__j	P_4__K	P_5__j	P_5__K	P_6__j	P_6__K	P_7__j	P_7__K	P_10__j	P_10__K
P_2__j	−18833.4	-	−583.7	87.0	−736.5	−5.3	−774.5	−2488.5	−2697.1	−2752.2	−2715.0	−2586.5	−2562.5	−2788.3	−2867.0
P_2__K	−18541.5	583.7	-	670.7	−152.8	578.4	−190.8	−1904.8	−2113.4	−2168.5	−2131.3	−2002.8	−1978.8	−2204.6	−2283.3
P_3__j	−18876.9	−87.0	−670.7	-	−823.5	−92.3	−861.5	−2575.5	−2784.1	−2839.2	−2802.0	−2673.5	−2649.5	−2875.3	−2954.0
P_3__K	−18465.1	736.5	152.8	823.5	-	731.2	−37.9	−1752.0	−1960.6	−2015.7	−1978.5	−1850.0	−1826.0	−2051.8	−2130.5
P_4__j	−18830.7	5.3	−578.4	92.3	−731.2	-	−769.2	−2483.2	−2691.8	−2746.9	−2709.7	−2581.2	−2557.2	−2783.0	−2861.7
P_4__K	−18446.2	774.5	190.8	861.5	37.9	769.2	-	−1714.0	−1922.6	−1977.7	−1940.5	−1812.0	−1788.1	−2013.9	−2092.6
P_5__j	−17589.1	2488.5	1904.8	2575.5	1752.0	2483.2	1714.0	-	−208.6	−263.7	−226.5	−98.0	−74.0	−299.8	−378.5
P_5__K	−17484.8	2697.1	2113.4	2784.1	1960.6	2691.8	1922.6	208.6	-	−55.1	−17.9	110.6	134.6	−91.2	−170.0
P_6__j	−17457.3	2752.2	2168.5	2839.2	2015.7	2746.9	1977.7	263.7	55.1	-	37.2	165.7	189.7	−36.1	−114.8
P_6__K	−17475.9	2715.0	2131.3	2802.0	1978.5	2709.7	1940.5	226.5	17.9	−37.2	-	128.5	152.5	−73.3	−152.0
P_7__j	−17540.1	2586.5	2002.8	2673.5	1850.0	2581.2	1812.0	98.0	−110.6	−165.7	−128.5	-	24.0	−201.8	−280.5
P_7__K	−17552.1	2562.5	1978.8	2649.5	1826.0	2557.2	1788.1	74.0	−134.6	−189.7	−152.5	−24.0	-	−225.8	−304.5
P_10__j	−17439.2	2788.3	2204.6	2875.3	2051.8	2783.0	2013.9	299.8	91.2	36.1	73.3	201.8	225.8	-	−78.7
P_10__K	−17399.9	2867.0	2283.3	2954.0	2130.5	2861.7	2092.6	378.5	170.0	114.8	152.0	280.5	304.5	78.7	-

ln *L* of each partition_model strategy and 2 ln Bayes factor results of comparisons between each partition_model strategy are given. The numerical subscripts next to the capital P mean the number of data partitions. The letter “j” and “K” mean the selected substitution model with jModeltest2 and Kakusan4, respectively.

### Phylogenetic relationships

Phylogenetic analyses employing BI and ML methods yielded identical topologies for the main clades and only minor differences at the terminals (shown in Figure 3). The monophyly of *Percocypris* was strongly supported in the results of all analyses. In addition, *Schizothorax* was recovered as the sister group of *Percocypris* in all our analyses. According to the topology (Figure 3) generated in this study, four deeply divergent major clades were identified as follows:

Clade A contained individuals that occur in Upper Yangtze River and formed a monophyletic clade with strong support (PP  = 1.00, BP  = 100%). This clade was recovered as the sister group of Clade B (PP  = 1.00, BP  = 100%).

In Clade B, all the individuals of *Percocypris regani* collected from Fuxian Lake clustered together with strong support (1.00 nodal support of PP and 100% of BP, respectively). The sample identified as “m36_NP” (Figure 3) collected from Upper Pearl River was recovered as the sister taxon of those from Fuxian Lake (PP  = 1.00, BP  = 100%).

All specimens collected from Mekong River constituted Clade C, which was a well supported monophyletic clade (PP  = 1.00, BP  = 100%).

Clade D included all individuals collected from Salween River and was well supported (PP  = 1.00, BP  = 100%). This clade was recovered as the sister group of clade C (PP  = 1.00, BP  = 100%).

In all cases, individuals from the same drainage clustered together with strong support. All of the analyses recovered the A + B clade as the sister group of the C + D clade with strong support, that is, the dichotomy between the two major clades was obvious in all the tree topologies.

### Divergence time and ancestral drainage

The combined result of the two independent chains showed effective sample size (ESS) value of posterior >200 (249.012) and ESS value of likelihood >4000 (4216.872). Large degrees of overlap in HPD interval were found between estimates of divergence times (Figure S1). *Percocypris* split from its sister group (*Schizothorax*) about 17.56 Mya (HPD 12.76–23.18) in the early Miocene. A little later, the split between Clade A + B and Clade C + D took place nearly in the same period (13.73 Mya; HPD 9.03–18.53). Subsequently, divergences within Clade A + B and within Clade C + D occurred at 5.1 Mya (HPD 2.82–7.92; the late Miocene/Pliocene) and 5.93 Mya (HPD 2.91–9.62; the Late Miocene/Pliocene), respectively. The apparent speciation event between populations of *Percocypris* from Fuxian Lake and those from Upper Pearl River took place about 2.16 Mya (HPD 1.03–3.71).

The result of ancestral drainage estimation carried out with primary BPA analysis is given in Table S5 and Figure S2. The area/species matrix for *Percocypris* is found in the Table S5 and the primary most parsimonious taxon/area cladogram in the Figure S2. The MRCA of the extant species of *Percocypris* probably inhabited a single paleo-drainage involving all the present-day drainages in which it is found. This hypothetical ancestor evolved into two major clades which were distributed in the paleo-drainages of contemporary Upper Yangtze River + Upper Pearl River and Mekong River + Salween River (for the MRCA of Clade A + B and Clade C + D, respectively). Furthermore, vicariance is the most parsimonious distribution hypothesis for the historical evolution of *Percocypris* suggested by primary BPA analysis (Figure S2); that is, the three MRCAs on the corresponding nodes probably evolved as a result of vicariant events (see the discussion section below).

## Discussion

This study represents the first phylogenetic hypothesis of the relationships of the species of *Percocypris*, including all taxa except for *P. tchangi* from Vietnam. The monophyly of the genus is strongly supported in this study; the placement of *Percocypris* as the sister group of *Schizothorax* is tested and supported, and the intrarelationships within *Percocypris* are assessed.

### Phylogenetic relationships and systematic implications for *Percocypris*

In our phylogenetic topology, the nominal *P. pingi* and *P. regani* clustered together as sister taxa with strong support. The average genetic distance between these two clades was 0.055 in Cyt *b* dataset and 0.026 in the combined dataset; this distance is equivalent to that between some recognized species of *Schizothorax*. In addition, the plots of these two clades in the PCA (Figure 4) resulted in non-overlapping, although adjacent, clusters. Considering the results of the average genetic distance and the PCA scatter plots, we support Kottelat's [8] suggestion that these two clades be raised to the species-level.

The PCA analysis (Figure 4) indicates that the specimens from Fuxian Lake and Upper Pearl River were clearly assignable to two distinct clusters. The divergence in topology and the distinct differences in morphological characters suggest that these two populations should be recognized as two distinct species (*P. regani* and a putative new species *P.* sp1), despite the fact that the genetic mean distance between these two clades is small (0.017 in Cyt *b* dataset and 0.008 in combined gene dataset).

For the nominal *P. retrodorslis* (Clades C and D), the samples are divided into two well-supported clades (i.e., Clade C from Mekong River and Clade D from Salween River). There is an average genetic distance of 0.063 in the Cyt *b* dataset and 0.029 in the combined gene dataset between these two clades, which is greater than the average genetic distances between some recognized species of *Schizothorax*. This suggests that the two clades should be treated as separate species. In addition, two distinct groups (Figure 4) were recognizable morphologically, which corresponded to the specimens from Mekong (Clade C) and Salween (Clade D) rivers. We found differences between these two clades in the skeletal system as follows: The insertion position of the first proximal pterygiophore of the dorsal fin is between the neural spines of the eighteenth and the nineteenth vertebral column in the individuals from Salween River, whereas for the individuals from Mekong River the position is between the neural spines of the seventeenth and eighteenth, or sixteenth and seventeenth vertebral column. Therefore, we conclude that there are two distinct species present, one in each river. We again follow Kottelat [8] in according *P. retrodorslis* specific status, and regard the Salween population as a putative new species (*P*. sp2).

In this study, we failed to obtain samples of *P. tchangi*, although we have sampled Red River in both Yunnan and Laocai (the type locality in Vietnam) on numerous occasions since 2003. In fact, there are no records of *P. tchangi* since the original description by Pellegrin and Chevey [9] in 1936. According to the description of the type specimen of *P. tchangi*, the position of dorsal fin is posteriorly situated, and a lateral stripe and scattered spots are present on the sides of the body. Further differences between *P. tchangi* and the other species of the genus are the number of lateral line scales and the body colour. The former is recorded as 60 [9], which is more than has been found in the other species (51–58). Furthermore, the upper body is brown and reddish, the lower body, upper head and the back are also reddish, and the fins are greyish and reddish [9]. The coloration of other species of the genus differs from *P. tchangi* in having a dark (black to brown) back and a blackish head; fins are blackish or orangish, and the lower body is yellowish (in formalin-fixed specimens). However, the original description of the type specimen of *P. tchangi* is not detailed enough, and the morphometric data is not accurate enough, to allow us to confidently place this species. There is no definitive evidence indicating that *P. retrodorslis* is a junior synonym of *P. tchangi* as suggested by Kottelat [8]. We provisionally regard both *P. retrodorslis* and *P. tchangi* as valid species. To confirm the placement of *P. tchangi*, the sample of this species should be included in future research.

In conclusion, our results support the discovery of two putative new species that need to be formally described. Therefore, we suggest that *Percocypris*, which should be reclassified as we propose above, appears to be a monophyletic group of six species: (1) *P. pingi* from Upper Yangtze River; (2) *P. regani* from Fuxian Lake; (3) *P.* sp1 (putative new species) from Upper Pearl River; (4) *P. retrodorslis* from Mekong River; (5) *P*. sp2 (putative new species) from Salween River; and (6) *P. tchangi* from Red River.

### The origin and evolutionary scenario of *Percocypris*

As shown below, *Percocypris* offers an excellent system for testing the hypotheses of the morphologies of the paleo-drainage basins of the Southeastern Tibetan Plateau, and the concomitant influences on the speciation of organisms living there.

**Figure 5:**
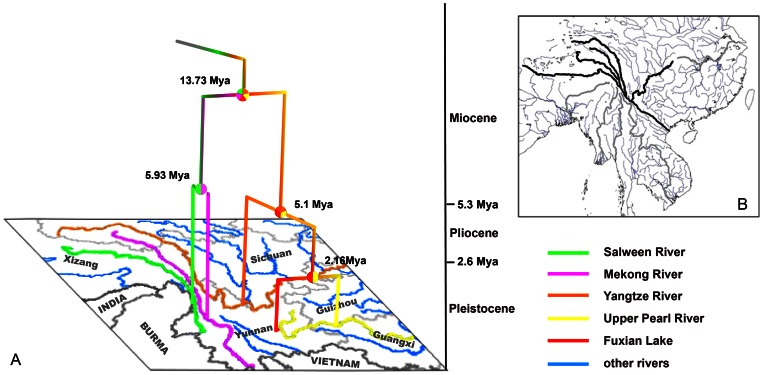
The origin and evolutionary scenario of *Percocypris*. Map (A) shows the time tree mapped on to the geography of southeast Qinhai-Tibetan Plateau. The nodal numbers are divergence times; the node circles show the ancestral drainages. Map (B) from Rüber et al. [14] show the hypothesized paleo-Red River drainage system of Clark et al. [60].

The results of divergence time and ancestral drainage estimations indicate that *Percocypris* probably originated in the early Miocene (17.56 Mya; Figure 5) from a single paleo-drainage that included current Upper Yangtze, Mekong, Salween, Upper Pearl, and probably Red rivers; this supports the hypothesis that original Upper Yangtze, Middle Yangtze, Upper Mekong and Upper Salween rivers drained together as major tributaries of the paleo-Red River drainage system [60]. Regarding the origin of *Percocypris*, it is noteworthy that our results strongly suggest that it may originate from a common ancestor with *Schizothorax*. This result is compatible with the hypothesis that *Percocypris* originated from a common ancestor with certain species of the Barbinae (e.g., [4]–[7]). The estimated divergence time of *Percocypris* and *Schizothorax* falls within the time range of the second uplift of the Tibetan Plateau (25–17 Mya; [61]–[64]).

Subsequently, the first diversification in *Percocypris* was the splitting into two main clades (“Clade A + Clade B” and “Clade C + Clade D”). We estimate this event to have occurred about 13.73 Mya (Figure 5), which is compatible with the earliest initiation age of rapid fluvial erosion in eastern Tibetan Plateau (13 Mya; [65]), presumably in response to the uplift of the Eastern Tibeten Plateau [65]. The results of primary BPA analysis show that the common ancestor of *P. retrodorslis* and *P*. sp2 occurred in a paleo-drainage of current Mekong and Salween rivers, and this inference is compatible with the hypothesis that these two rivers were once connected, with Salween River as a tributary of Mekong River [60]. Additionally, our estimated divergence time of *P*. *retrodorslis* and *P*. sp2 (5.93 Mya; “Clade C + D”) occurred during the Late Miocene/Early Pliocene. This timing supports the hypothesis that Salween River started to form since the Middle and Late Miocene [66]. Furthermore, the hypothesis of an ancestral drainage of connecting contemporary Upper Yangtze and Upper Pearl rivers, where the common ancestor of *P. pingi*, *P. regani* and *P*. sp1 is hypothesized to have occurred, may imply that these two drainages were joined for some time. The separations of these species are consistent with the changes in river patterns [60], [67]. According to the divergence time estimation of the node of *P. regani* and *P*. sp1, the split between these two species (2.16 Mya; Figure 5) seems to be in approximate agreement with the time of formation of Fuxian Lake during the Pliocene (3.0–3.4 Mya; [68]).

Thus, based on primary BPA analysis, the paleo-drainage of all current drainage basins split initially into two paleo-drainages (i.e., one containing Upper Yangtze River and Upper Pearl River; the other containing Mekong and Salween rivers). This could be considered as vicariant events (Figure S2). The primary BPA analysis suggests that two additional vicariant events in the speciation of *Percocypris* occurred after the split of the two paleo-drainages (Figure S2). These were the speciation of *P. pingi* and *P. regani*-*P*. sp1 by the isolation between Upper Yangtze River and Upper Pearl River, and the speciation of *P. retrodorslis* and *P*. sp2 by the separation of Mekong and Salween rivers. According to our estimated separation time, the first vicariant event most likely occurred during the initiation age of rapid river erosion and capture in eastern Tibetan Plateau, and the subsequent vicariant events of Mekong and Salween rivers appear compatible with the formation of Salween River. The fluvial erosion and river capture leading to isolation events in *Percocypris* presumably reacted to the uplift of the Southeastern Tibetan Plateau during the Miocene [65], [66].

Large paleo-drainages may have acted as barriers to terricolous animals (e.g. *Nanorana yunnanensis* [69]; *Apodemus ilex* [70]) and plants (e.g. *Terminalia franchetii*; [71], [72]), which invoked the paleo-Red River hypothesis [60]. The paleo-Red River hypothesis was also tested by other fish biogeographic studies (e.g. Badidae [14]; Glyptosternoidae [15]; Sisoridae [16]; *Schizothorax* [17], [73]). However, the divergence time of *Schizothorax* of Upper Yangtze River and Mekong River + Salween River (6.8 Mya –6.2 Mya; [17]) was much later than those in *Percocypris* (13.73 Mya; Figure 5). This may imply that Upper Yangtze River and Mekong River + Salween River were connected for some time by small-scale tributaries after the main river split. The discrepancy of the divergence times may be attributable to the different ecological niche between *Schizothorax* and *Percocypris*. *Percocypris* inhabit large bodies of water (large rivers or lakes); smaller bodies of water seem to act as barriers to the species of *Percocypris*. Therefore, *Percocypris* should be sensitive to the change of large-scale morphologies of paleo-drainages basins.

In summary, this study indicates that geological vicariance based on the changes in the large-scale morphologies of the paleo-drainage basins in the Southeastern Tibetan Plateau has played an important role in the speciation of *Percocypris*. The biogeographic relationships of *Percocypris* (as shown in Figure 5) could test the paleo-Red River hypothesis more simply and clearly. With the specific distribution pattern of only one species in each drainage and specific ecological niche, *Percocypris* seem to be an ideal system for testing the hypotheses of the morphologies of the paleo-drainage basins of the Southeastern Tibetan Plateau. Clearly, similar biogeographic results for additional taxa and detailed paleogeographic evidences are needed to fully understand the influence of the paleo-drainage basin morphologies surrounding the Southeastern Tibetan Plateau on the speciation of the organisms inhabiting this area.

### Trait divergence in *Percocypris* from lake and river

Two main habitat types are found among the species of *Percocypris* in this study: (1) the lake type of *P. regani* in Fuxian Lake; (2) the river type of *P*. sp1, *P. pingi P. retrodorslis* and *P*. sp2 inhabiting Upper Pearl, Upper Yangtze, Mekong and Salween rivers. As the result of the PCA indicates (Figure 4), the individuals from the lake habitat (Fuxian Lake) formed a separate cluster from those from the riverine habitats (Upper Pearl, Upper Yangtze, Mekong and Salween rivers). The differences were reflected in the length of the fins, the distance between the origin of each fin base and the snout, the proportions of the head (head depth and head width) and the length of barbels (maxillary and rictal barbels).

Morphological divergence associated with lentic and lotic habitat was observed in many kinds of fishes. Different species reflect different aspects of traits divergence. In the well-investigated threespine stickleback (*Gasterosteus aculeatus*) it was found that the lake ecotypes displayed more developed gill structures (more numerous gill rakers) and more streamlined bodies (deeper bodies, shorter pelvic, dorsal spines, deeper caudal peduncles) than the stream counterparts [74]–[83]. Two Neotropical fish (*Bryconops caudomaculatus*; *Biotodoma wavrini*) showed morphological divergence in of maximum body depth and mouth orientation between lotic channels and lentic lagoons [84]. *Cyprinella venusta* in rivers and reservoirs exhibited differences in the proportions of the head, the position of eye, the position of dorsal fin and the length of dorsal fin base [85]. In the study of the rainbow fishes (*Melanotaenia eachamensis* and *M. duboulayi*) [86], trait divergence between lake and stream habitats was found in the position first dorsal and pelvic fins, the length of second dorsal fin bases. The lentic–lotic divergences in morphological traits have been observed in many other fish (e.g. *Cyprinella lutrensis*, [87]; *Phoxinus phoxinus*, [88]; *Oncorhynchus mykiss*, [89]; *Oncorhynchus nerka*, [90]; *Osteochilus hasseltii*, [91]).

In this study, *P. regani* inhabiting lakes have more anterior fins and smaller heads than the other species of *Percocypris* inhabiting rivers. The divergence of the position of the dorsal fin could also be observed in the number of neural spines before the pterygiophore (see the result of bivariate correlation analysis). These findings are congruent with the morphological differences between reservoirs and rivers observed in the two species of *Cyprinella* (*C. venusta*, [85]; *C. lutrensis*, [87]). Cui & Chu [6] suggested that the posterior placement of the dorsal fin may be an adaptive trait related to the predatory nature of the species of this genus. In addition, we found that the positions of the fins (except the caudal fin) may also be related to the habitat type; that is, the position of each fin is more posteriorly situated in the species from the rivers than those from the lake. Strikingly, compared with the rainbow fishes *Melanotaenia eachamensis* and *M. duboulayi* (McGuigan et al. [86]), the dorsal fin position of *Percocypris* appears to diverge in the opposite direction relative to lentic and lotic habitat. The rainbow fishes in the lake had a more posteriorly positioned first dorsal fin than those in the streams [86]. McGuigan et al. [86] hypothesized that the posterior shift in the first dorsal fin of the rainbow fish was driven by selection with the change of different water velocity habitat, but they could not provide firm evidence that selection drove the evolution of the relative fin positions in their system. The effect of water velocity on the position of pterygiophore/dorsal fin and associated traits might be highly variable in different systems. Further and deeper ecological and kinematic studies may help to elucidate the correlation between the water velocity and the position of pterygiophore/dorsal fin. Cui & Chu [6] hypothesized that the narrow head enabled broader vision in *P. regani*, which provided an advantage while hunting in the clearer waters of Fuxian Lake. In the stickleback, changes in head size and eye position may be related to the shifts in prey type [92]. The head of *Percocypris* inhabiting the rivers with turbid water was wider than those in the lake, and this may indicate that vision is less important in prey acquisition in this environment. The maxillary and rictal barbels of *Percocypris* in rivers are longer than those in the lake, and this may also be correlated with more limited vision in the turbid waters of the rivers. In addition, for the differences in the lengths of fins between lacustrine and riverine species, we support the hypothesis that riverine fishes have longer pectoral, anal and dorsal fins for the stability and manoeuvrability in the water flow [93], [94]. Drinan et al. [95] found that *Salmo trutta* from high-gradient (rapid water flow) rivers have longer pectoral fins than those from low-gradient rivers to increase stability and manoeuvrability.

### Conservation implications for genus *Percocypris*

The IUCN Red List for China lists *P. pingi* (using Cui & Chu's [6] arrangement of three subspecies) as “Vulnerable” (VU). However, according to the results of our phylogenetic analyses, *Percocypris* should be reclassified as six species. Our suggestion of this reclassification may be helpful in developing conservation strategies for the species of this genus, based on the views of Amato & Schaller [96] and Vogler & DeSalle [97] that phylogenetic information can provide data useful for prioritizing conservation strategies. Within this new classificatory framework, the conservation status of each of the six species of *Percocypris* requires reassessment. Therefore, conservation efforts should be directed to the six species and their relevant habitats.

The fishes of Upper Yangtze, Upper Pearl, Mekong, Salween and Red rivers have been extensively sampled since 1977; our field records covering several decades and the information provided by local people show that populations of all of the species of *Percocypris* have decreased in recent years.

*Percocypris pingi* in Upper Yangtze River, *P. regani* in Fuxian Lake, *P. retrodorslis* in Mekong River and *P*. sp2 in Salween River were difficult to find, especially in recent years, as our field records and the information from the local people demonstrate. Even worse, the number of *P*. sp1 in Upper Pearl River is very low probably due to pollution produced by heavy-metal enterprises along the river. Noticeably, *P. tchangi* in Red River has not been found since the species was described in 1936, a period of 77 years. Unfortunately, the species of this genus are threatened due to habitat destruction by water pollution as well as other factors such as overfishing and illegal fishing. As predators of other fishes, the species of *Percocypris* are keystone species in the relevant drainages, which have a significant impact on the maintenance of the ecological community structure. As a group of highly endemic species, immediate specific conservation strategies and additional studies on conservation for this genus are urgently needed.

## Supporting Information

Figure S1 **The results of divergence time using the Bayesian relaxed clock method (A). Chronogram of** ***Percocypris*** **(B).**
(TIF)

Figure S2 **Primary BPA taxa/area cladogram of** ***Percocypris*** **.** A – Upper Yangtze River, B – Upper Pearl River, C – Fuxian Lake, D – Mekong River, E – Salween River. The numbers “1–9” refer to Table S5.
(TIF)

Table S1 **Samples of species, with voucher number, locality and drainage information for the specimens sampled, including the GenBank accession numbers.**
(DOC)

Table S2 **PCR primers (names, sequences and references) to amplify three mitochondrial and one nuclear DNA genes.**
(DOC)

Table S3 **Summary of partitioned substitution models (BIC) for the phylogenetic analyses and divergence time estimation.**
(DOC)

Table S4 **The statistics of the counts of meristic characters and osteological traits.**
(DOC)

Table S5 **Primary BPA matrix listing the distribution of** ***Percocypris*** **, along with the binary codes representing the phylogenetic relationships among the genus.**
(DOC)
