# Supplementary figures and images for: Molecular Phylogeny and Biogeography of Percocypris (Cyprinidae, Teleostei)
Source: PLoS One. 2013 Jun 4;8(6):e61827. doi: 10.1371/journal.pone.0061827 (PMC3672144; doi:10.1371/journal.pone.0061827)

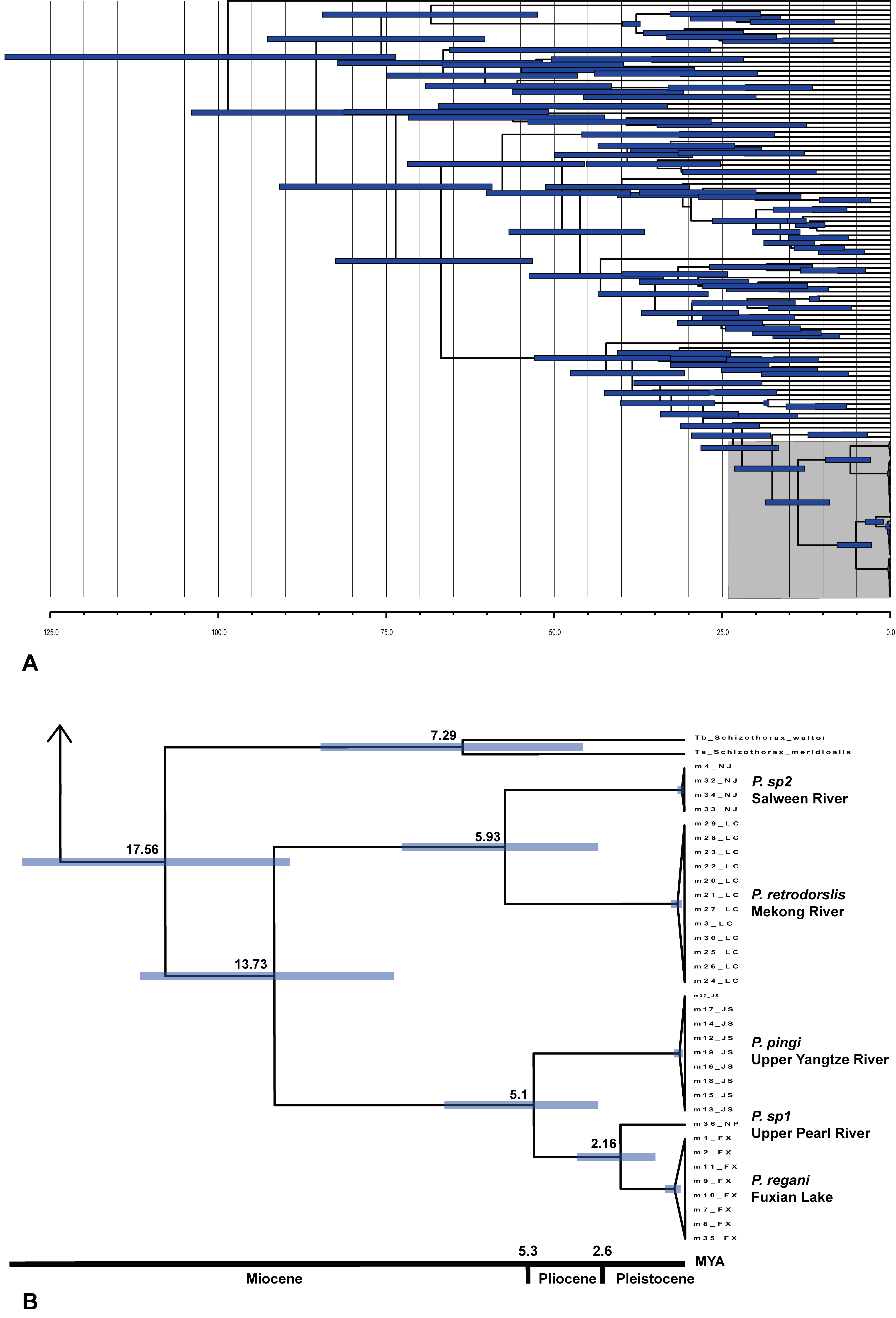

Supplement: Figure S1 — The results of divergence time using the Bayesian relaxed clock method (A). Chronogram of Percocypris (B). (TIF) [file pone.0061827.s001.tif]

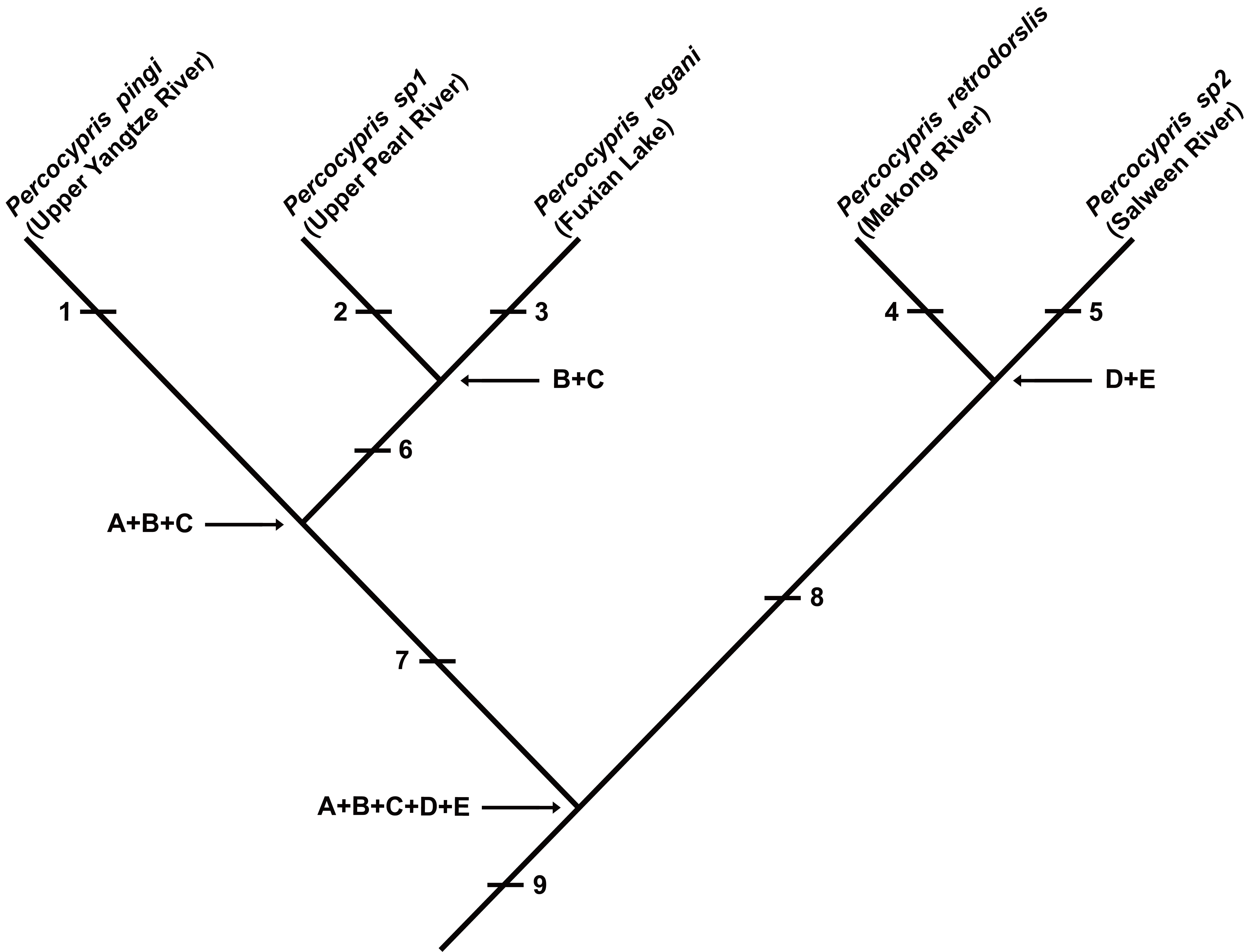

Supplement: Figure S2 — Primary BPA taxa/area cladogram of Percocypris . A – Upper Yangtze River, B – Upper Pearl River, C – Fuxian Lake, D – Mekong River, E – Salween River. The numbers “1–9” refer to Table S5. (TIF) [file pone.0061827.s002.tif]
