# Supplementary material for: Molecular Phylogeny and Biogeography of Percocypris (Cyprinidae, Teleostei)
Source: PLoS One. 2013 Jun 4;8(6):e61827. doi: 10.1371/journal.pone.0061827 (PMC3672144; doi:10.1371/journal.pone.0061827)
Supplement: Table S1 — Samples of species, with voucher number, locality and drainage information for the specimens sampled, including the GenBank accession numbers. (DOC) [file pone.0061827.s003.doc]

Table S1. Samples of species, with voucher number, locality and drainage information for the specimens sampled, including the GenBank accession numbers.

| **Family** | **Species** | **Collection location Locality** | **Drainage** | **GenBank accession numbers** | | | |
| --- | --- | --- | --- | --- | --- | --- | --- |
|  |  |  |  | **16S** | **COI** | **Cyt *b*** | **Rag2** |
| **Cyprinidae** |  |  |  |  |  |  |  |
|  | **Ingroups** |  |  |  |  |  |  |
|  | *P. regani ^1, 2^* | Chengjiang, Yunnan Prov. | Upper Pearl River (Fuxian Lake) | JX042095* | JX042132* | JX042169* | JX042058* |
|  | *P. regani ^1, 2^* | Chengjiang, Yunnan Prov. | Upper Pearl River (Fuxian Lake) | JX042096* | JX042133* | JX042170* | JX042059* |
|  | *P. regani ^1, 2^* | Chengjiang, Yunnan Prov. | Upper Pearl River (Fuxian Lake) | JX042099* | JX042136* | JX042173* | JX042062* |
|  | *P. regani ^1, 2^* | Chengjiang, Yunnan Prov. | Upper Pearl River (Fuxian Lake) | JX042100* | JX042137* | JX042174* | JX042063* |
|  | *P. regani ^1, 2^* | Chengjiang, Yunnan Prov. | Upper Pearl River (Fuxian Lake) | JX042101* | JX042138* | JX042175* | JX042064* |
|  | *P. regani ^1, 2^* | Chengjiang, Yunnan Prov. | Upper Pearl River (Fuxian Lake) | JX042102* | JX042139* | JX042176* | JX042065* |
|  | *P. regani ^1, 2^* | Chengjiang, Yunnan Prov. | Upper Pearl River (Fuxian Lake) | JX042103* | JX042140* | JX042177* | JX042066* |
|  | *P. regani ^1, 2^* | Chengjiang, Yunnan Prov. | Upper Pearl River (Fuxian Lake) | JX042126* | JX042163* | JX042200* | JX042089* |
|  | *P.* sp1 *^1, 2^* | Luoping, Yunnan Prov. | Upper Pearl River | JX042127* | JX042164* | JX042201* | JX042090* |
|  | *P. pingi ^1, 2^* | Yongren, Yunnan Prov. | Upper Yangtze River | JX042104* | JX042141* | JX042178* | JX042067* |
|  | *P. pingi ^1, 2^* | Yongren, Yunnan Prov. | Upper Yangtze River | JX042105* | JX042142* | JX042179* | JX042068* |
|  | *P. pingi ^1, 2^* | Yongren, Yunnan Prov. | Upper Yangtze River | JX042106* | JX042143* | JX042180* | JX042069* |
|  | *P. pingi ^1, 2^* | Panzhihua, Sichuan Prov. | Upper Yangtze River | JX042107* | JX042144* | JX042181* | JX042070* |
|  | *P. pingi ^1, 2^* | Panzhihua, Sichuan Prov. | Upper Yangtze River | JX042108* | JX042145* | JX042182* | JX042071* |
|  | *P. pingi ^1, 2^* | Panzhihua, Sichuan Prov. | Upper Yangtze River | JX042109* | JX042146* | JX042183* | JX042072* |
|  | *P. pingi ^1, 2^* | Panzhihua, Sichuan Prov. | Upper Yangtze River | JX042110* | JX042147* | JX042184* | JX042073* |
|  | *P. pingi ^1, 2^* | Yongren, Yunnan Prov. | Upper Yangtze River | JX042111* | JX042148* | JX042185* | JX042074* |
|  | *P. pingi ^1, 2^* | Yulong, Yunnan Prov. | Upper Yangtze River | JX042128* | JX042165* | JX042202* | JX042091* |
|  | *P.* *retrodorslis ^1, 2^* | Yunlong, Yunnan Prov. | Mekong River | JX042097* | JX042134* | JX042171* | JX042060* |
|  | *P. retrodorslis ^1, 2^* | Fengqing, Yunnan Prov. | Mekong River | JX042112* | JX042149* | JX042186* | JX042075* |
|  | *P. retrodorslis ^1, 2^* | Gengma, Yunnan Prov. | Mekong River | JX042113* | JX042150* | JX042187* | JX042076* |
|  | *P. retrodorslis ^1, 2^* | Gengma, Yunnan Prov. | Mekong River | JX042114* | JX042151* | JX042188* | JX042077* |
|  | *P. retrodorslis ^1, 2^* | Gengma, Yunnan Prov. | Mekong River | JX042115* | JX042152* | JX042189* | JX042078* |
|  | *P. retrodorslis ^1, 2^* | Baoshan, Yunnan Prov. | Mekong River | JX042116* | JX042153* | JX042190* | JX042079* |
|  | *P. retrodorslis ^1, 2^* | Baoshan, Yunnan Prov. | Mekong River | JX042117* | JX042154* | JX042191* | JX042080* |
|  | *P. retrodorslis ^1, 2^* | Yunlong, Yunnan Prov. | Mekong River | JX042118* | JX042155* | JX042192* | JX042081* |
|  | *P. retrodorslis ^1, 2^* | Yunlong, Yunnan Prov. | Mekong River | JX042119* | JX042156* | JX042193* | JX042082* |
|  | *P. retrodorslis ^1, 2^* | Yunlong, Yunnan Prov. | Mekong River | JX042120* | JX042157* | JX042194* | JX042083* |
|  | *P. retrodorslis ^1, 2^* | Yunlong, Yunnan Prov. | Mekong River | JX042121* | JX042158* | JX042195* | JX042084* |
|  | *P. retrodorslis ^1, 2^* | Yunlong, Yunnan Prov. | Mekong River | JX042122* | JX042159* | JX042196* | JX042085* |
|  | *P.* sp2 *^1, 2^* | Longling, Yunnan Prov. | Salween River | JX042098* | JX042135* | JX042172* | JX042061* |
|  | *P.* sp2 *^1, 2^* | Longling, Yunnan Prov. | Salween River | JX042123* | JX042160* | JX042197* | JX042086* |
|  | *P.* sp2 *^1, 2^* | Longling, Yunnan Prov. | Salween River | JX042124* | JX042161* | JX042198* | JX042087* |
|  | *P.* sp2 *^1, 2^* | Longling, Yunnan Prov. | Salween River | JX042125* | JX042162* | JX042199* | JX042088* |
|  | **Outgroups** |  |  |  |  |  |  |
|  | *Schizothorax meridionalis ^1, 2^* |  |  | DQ845847 | —— | AY954285 | DQ366989 |
|  | *Schizothorax waltoni ^1, 2^* |  |  | HM536761 | HM536903 | HM536804 | DQ366981 |
|  | *Onychostoma simum ^1, 2^* |  |  | DQ845861 | HM536899 | HM536801 | DQ366991 |
|  | *Sinocyclocheilus tingi ^1, 2^* |  |  | DQ845866 | —— | AY854701 | DQ366978 |
|  | *Barbus barbus ^2^* |  |  | AB238965 | AB238965 | AB238965 | —— |
|  | *Luciobarbus capito* *^2^* |  |  | JX987313 | JX987313 | JX987313 | —— |
|  | *Capoeta capoeta ^2^* |  |  | EU707354 | JQ623921 | JF798331 | —— |
|  | *Gymnocypris przewalskii ^2^* |  |  | AB239595 | AB239595 | AB239595 | —— |
|  | *Spinibarbus denticulatus ^1, 2^* |  |  | JX042131* | JX042168* | JX042205* | JX042094* |
|  | *Cyprinus pellegrini ^1, 2^* |  |  | JX042129* | JX042166* | JX042203* | JX042092* |
|  | *Carassius auratus ^1, 2^* |  |  | AB111951 | AB111951 | AB111951 | DQ366941 |
|  | *Barbonymus schwanenfeldii ^1, 2^* |  |  | DQ845906 | —— | AF180823 | DQ366961 |
|  | *Puntius ticto ^2^* |  |  | AB238969 | AB238969 | AB238969 | —— |
|  | *Rectoris posehensis ^2^* |  |  | GU168756 | GU086595 | GU086557 | —— |
|  | *Ptychidio jordani ^2^* |  |  | GU168741 | GU086580 | GU086542 | —— |
|  | *Discogobio brachyphysallidos ^2^* |  |  | GU168746 | GU086585 | GU086547 | —— |
|  | *Cirrhinus molitorella ^2^* |  |  | GU168737 | GU086576 | GU086538 | —— |
|  | *Osteochilus salsburyi ^2^* |  |  | GU168738 | GU086577 | GU086539 | —— |
|  | *Garra orientalis ^2^* |  |  | GU168763 | GU086602 | GU086564 | —— |
|  | *Labeo stoliczkae ^1, 2^* |  |  | JX042130* | JX042167* | JX042204* | JX042093* |
|  | *Tor douronensis ^1, 2^* |  |  | DQ845877 | —— | FJ211162 | DQ366945 |
|  | *Culter mongolicus* *^2^* |  |  | AP009060 | AP009060 | AP009060 | —— |
|  | *Megalobrama terminalis ^2^* |  |  | NC_018816 | NC_018816 | NC_018816 | —— |
|  | *Ischikauia steenackeri ^2^* |  |  | AB239601 | AB239601 | AB239601 | —— |
|  | *Xenocypris argentea* *^2^* |  |  | AP009059 | AP009059 | AP009059 | —— |
|  | *Distoechodon tumirostris ^2^* |  |  | DQ026431 | DQ026431 | DQ026431 | —— |
|  | *Mylopharyngodon piceus* *^2^* |  |  | DQ026435 | DQ026435 | DQ026435 | —— |
|  | *Ctenopharyngodon idella ^2^* |  |  | NC_010288 | NC_010288 | NC_010288 | —— |
|  | *Hypophthalmichthys molitrix* *^2^* |  |  | NC_010156 | NC_010156 | NC_010156 | —— |
|  | *Squaliobarbus curriculus ^2^* |  |  | JX910141 | JX910141 | JX910141 | —— |
|  | *Ochetobius elongatus ^2^* |  |  | GQ406275 | —— | AF309506 | —— |
|  | *Elopichthys bambusa ^2^* |  |  | GQ406274 | —— | GQ406332 | —— |
|  | *Hemigrammocypris rasborella ^2^* |  |  | AP011422 | AP011422 | AP011422 | —— |
|  | *Opsariichthys bidens ^2^* |  |  | DQ367044 | DQ367044 | DQ367044 | —— |
|  | *Opsariichthys uncirostris ^2^* |  |  | AB218897 | AB218897 | AB218897 | —— |
|  | *Zacco sieboldii ^2^* |  |  | AB218898 | AB218898 | AB218898 | —— |
|  | *Aphyocypris chinensis ^2^* |  |  | AB218688 | AB218688 | AB218688 | —— |
|  | *Tinca tinca ^2^* |  |  | AB218686 | AB218686 | AB218686 | —— |
|  | *Cyprinella lutrensis* *^2^* |  |  | AB070206 | AB070206 | AB070206 | —— |
|  | *Notropis stramineus ^2^* |  |  | NC_008110 | NC_008110 | NC_008110 | —— |
|  | *Pimephales promela* *^2^* |  |  | GQ406272 | FJ918908 | GQ184520 | —— |
|  | *Phenacobius mirabilis ^2^* |  |  | NC_008112 | NC_008112 | NC_008112 | —— |
|  | *Rhinichthys atratulus* *^2^* |  |  | AF038495 | HQ557131 | AF452078 | —— |
|  | *Campostoma anomalum ^2^* |  |  | NC_008102 | NC_008102 | NC_008102 | —— |
|  | *Mylocheilus caurinus ^2^* |  |  | AP010779 | AP010779 | AP010779 | —— |
|  | *Pogonichthys macrolepidotus* *^2^* |  |  | AF081860 | —— | JX443009 | —— |
|  | *Clinostomus elongatus ^2^* |  |  | AF081840 | EU524487 | GU182822 | —— |
|  | *Richardsonius balteatus ^2^* |  |  | AF081861 | EF452858 | JX442994 | —— |
|  | *Pseudaspius lectocephalus ^2^* |  |  | AP009058 | AP009058 | AP009058 | —— |
|  | *Tribolodon nakamurai ^2^* |  |  | AB218896 | AB218896 | AB218896 | —— |
|  | *Phoxinus perenurus ^2^* |  |  | AP009061 | AP009061 | AP009061 | —— |
|  | *Pelecus clutratus ^2^* |  |  | AB239597 | AB239597 | AB239597 | —— |
|  | *Notemigonus crysoleucas ^2^* |  |  | AB127393 | AB127393 | AB127393 | —— |
|  | *Alburnus alburnus ^2^* |  |  | AB239593 | AB239593 | AB239593 | —— |
|  | *Leuciscus leuciscus ^2^* |  |  | GQ406268 | HQ961025 | HM560100 | —— |
|  | *Tanichthys albonubes ^2^* |  |  | AP011397 | AP011397 | AP011397 | —— |
|  | *Gobiobotia brevibarba ^2^* |  |  | FJ515919 | FJ515919 | FJ515919 | —— |
|  | *Pseudogobio esocinus ^2^* |  |  | AP009310 | AP009310 | AP009310 | —— |
|  | *Hemibarbus barbus ^2^* |  |  | AB070241 | AB070241 | AB070241 | —— |
|  | *Gobio gobio ^2^* |  |  | AB239596 | AB239596 | AB239596 | —— |
|  | *Gnathopogon elongates ^2^* |  |  | AB218687 | AB218687 | AB218687 | —— |
|  | *Sarcocheilichthys variegatus ^2^* |  |  | AB054124 | AB054124 | AB054124 | —— |
|  | *Pseudorasbora pumila pumila ^2^* |  |  | AB239599 | AB239599 | AB239599 | —— |
|  | *Pungtungio herzi ^2^* |  |  | AB239598 | AB239598 | AB239598 | —— |
|  | *Acheilognathus typus ^2^* |  |  | AB239602 | AB239602 | AB239602 | —— |
|  | *Rhodeus ocellatus kurumeus ^2^* |  |  | AB070205 | AB070205 | AB070205 | —— |
|  | *Raiamas senegalensis ^2^* |  |  | AP010780 | AP010780 | AP010780 | —— |
|  | *Barilius bendelisis ^2^* |  |  | AP011433 | AP011433 | AP011433 | —— |
|  | *Salmostoma bacaila ^2^* |  |  | AP011223 | AP011223 | AP011223 | —— |
|  | *Danio rerio ^1, 2^* |  |  | NC_002333 | NC_002333 | NC_002333 | NM_131385 |
|  | *Esomus metallicus ^2^* |  |  | AB239594 | AB239594 | AB239594 | —— |
|  | *Rasbora vaterifloris ^2^* |  |  | AP011432 | AP011432 | AP011432 | —— |
| **Balitoridae** |  |  |  |  |  |  |  |
|  | *Homaloptera leonardi ^2^* |  |  | AB242165 | AB242165 | AB242165 | —— |
|  | *Crossostoma lacustre ^2^* |  |  | M91245 | M91245 | M91245 | —— |
|  | *Barbatula toni* *^2^* |  |  | AB242162 | AB242162 | AB242162 | —— |
|  | *Lefua echigonia ^2^* |  |  | AB054126 | AB054126 | AB054126 | —— |
|  | *Schistura balteata ^2^* |  |  | AB242172 | AB242172 | AB242172 | —— |
|  | *Vaillantella maassi ^2^* |  |  | AB242173 | AB242173 | AB242173 | —— |
| **Cobitidae** |  |  |  |  |  |  |  |
|  | *Misgurnus nikolskyi ^2^* |  |  | AB242171 | AB242171 | AB242171 | —— |
|  | *Cobitis striata ^2^* |  |  | AB054125 | AB054125 | AB054125 | —— |
|  | *Pangio anguillaris ^2^* |  |  | AB242168 | AB242168 | AB242168 | —— |
|  | *Acantopsis choirorhynchos ^2^* |  |  | AB242161 | AB242161 | AB242161 | —— |
|  | *Leptobotia mantschurica ^2^* |  |  | AB242170 | AB242170 | AB242170 | —— |
|  | *Chromobotia macracanthus ^2^* |  |  | AB242163 | AB242163 | AB242163 | —— |
| **Catostomidae** |  |  |  |  |  |  |  |
|  | *Hypentelium nigricans ^2^* |  |  | AB242169 | AB242169 | AB242169 | —— |
|  | *Moxostoma poecilurum ^2^* |  |  | AB242167 | AB242167 | AB242167 | —— |
|  | *Minytrema melanops ^2^* |  |  | AB242166 | AB242166 | AB242166 | —— |
|  | *Catostomus commersonii ^2^* |  |  | AB127394 | AB127394 | AB127394 | —— |
|  | *Cycloptus elongates ^2^* |  |  | AB126082 | AB126082 | AB126082 | —— |
|  | *Carpiodes carpio ^2^* |  |  | AP006763 | AP006763 | AP006763 | —— |
|  | *Myxocyprinus asiaticus ^2^* |  |  | AP006764 | AP006764 | AP006764 | —— |
|  | *Ictiobus bubalus ^2^* |  |  | AP009316 | AP009316 | AP009316 | —— |
|  | *Gyrinocheilus aymonieri ^2^* |  |  | AB242164 | AB242164 | AB242164 | —— |
| **Callichthyidae** |  |  |  |  |  |  |  |
|  | *Corydoras rabauti ^2^* |  |  | AB054128 | AB054128 | AB054128 | —— |

The asterisks (*) mean the sequence obtain in this study. The numbers (^1, 2^) mean the sequence used for the phylogenetic analyses and divergence time estimation, respectively.
