# Supplementary material for: Molecular Phylogeny and Biogeography of Percocypris (Cyprinidae, Teleostei)
Source: PLoS One. 2013 Jun 4;8(6):e61827. doi: 10.1371/journal.pone.0061827 (PMC3672144; doi:10.1371/journal.pone.0061827)
Supplement: Table S2 — PCR primers (names, sequences and references) to amplify three mitochondrial and one nuclear DNA genes. (DOC) [file pone.0061827.s004.doc]

Table S2. PCR primers (names, sequences and references) to amplify three mitochondrial and one nuclear DNA genes.

| **Genes** | **Primer name** | **Primer sequence (5'-3')** | **Cited source** |
| --- | --- | --- | --- |
| 16S | 16Sp1F | CTT ACA CCG AGA ARA CAT C | Li et al. [1] |
|  | 16Sp1R | CTT AAG CTC CAA AGG GTC | Li et al. [1] |
| COI | COI-L5956 | CACAAAGACATTGGCACCCT | Miya & Nishida [2] |
|  | COI-H6855 | AGTCAGCTGAAKACTTTTAC | Miya & Nishida [2] |
| Cyt *b* | L14724 | GACTTGAAAAACCACCGTTG | Xiao et al. [3] |
|  | H15915 | CTCCGATCTCCGGATTACAAGAC | Xiao et al. [3] |
| Rag2 | RAG2-f2^a^ | ArACGCTCmTGTCCmACTGG | Lovejoy & Collette [4] |
|  | RAG2-R6^a^ | TGrTCCArGCAGAAGTACTTG | Lovejoy & Collette [4] |

References

1. Li J, Wang X, Kong X, Zhao K, He S, et al. (2008) Variation patterns of the mitochondrial 16S rRNA gene with secondary structure constraints and their application to phylogeny of cyprinine fishes (Teleostei: Cypriniformes). Mol Phylogenet Evol 47: 472-487.

2. Miya M, Nishida M (2000) Use of Mitogenomic Information in Teleostean Molecular Phylogenetics: A Tree-Based Exploration under the Maximum-Parsimony Optimality Criterion. Mol Phylogenet Evol 17: 437-455.

3. Xiao W, Zhang Y, Liu H (2001) Molecular Systematics of Xenocyprinae (Teleostei: Cyprinidae): Taxonomy, Biogeography, and Coevolution of a Special Group Restricted in East Asia. Mol Phylogenet Evol 18: 163-173.

4. Lovejoy NR, Collette BB, McEachran JD (2001) Phylogenetic Relationships of New World Needlefishes (Teleostei: Belonidae) and the Biogeography of Transitions between Marine and Freshwater Habitats. Copeia 2001: 324-338.
