# Supplementary material for: Molecular Phylogeny and Biogeography of Percocypris (Cyprinidae, Teleostei)
Source: PLoS One. 2013 Jun 4;8(6):e61827. doi: 10.1371/journal.pone.0061827 (PMC3672144; doi:10.1371/journal.pone.0061827)
Supplement: Table S3 — Summary of partitioned substitution models (BIC) for the phylogenetic analyses and divergence time estimation. (DOC) [file pone.0061827.s005.doc]

**Table S3.** **Summary of partitioned substitution models (BIC) for the phylogenetic analyses and** **divergence time estimation.**

| **Partition** | **For phylogenetic analyses** | | |
| --- | --- | --- | --- |
|  | **Model Selected** | **Nst** | **Rates** |
| 16S | GTR+G | 6 | gamma |
| COI_1 | SYM+G | 6 | gamma |
| COI_2 | F81+G | 1 | equal |
| COI_3 | HKY+G | 2 | gamma |
| Cyt *b*_1 | SYM+G | 6 | gamma |
| Cyt *b*_2 | HKY+G | 2 | gamma |
| Cyt *b*_3 | GTR+G | 6 | gamma |
| Rag2_1 | K80+G | 2 | gamma |
| Rag2_2 | K80+G | 2 | gamma |
| Rag2_3 | HKY+G | 2 | gamma |
| **Partition** | **For divergence time estimation** | | |
|  | **Model Selected** | **Nst** | **Rates** |
| 16S | GTR+G | 6 | gamma |
| COI_1 | SYM+G | 6 | gamma |
| COI_2 | HKY+G | 2 | gamma |
| COI_3 | GTR+G | 6 | gamma |
| Cyt *b*_1 | SYM+G | 6 | gamma |
| Cyt *b*_2 | HKY+G | 2 | gamma |
| Cyt *b*_3 | HKY+G | 2 | gamma |

The number (1 2 3) mean the first, second and third codon position for the COI, Cyt *b* and Rag2 gene, respectively.
