# Supplementary material for: Molecular Phylogeny and Biogeography of Percocypris (Cyprinidae, Teleostei)
Source: PLoS One. 2013 Jun 4;8(6):e61827. doi: 10.1371/journal.pone.0061827 (PMC3672144; doi:10.1371/journal.pone.0061827)
Supplement: Table S4 — The statistics of the counts of meristic characters and osteological traits. (DOC) [file pone.0061827.s006.doc]

Table S4. The statistics of the counts of meristic characters and osteological traits.

| **Characters** | **Fuxian Lake** | **Upper Pearl River** | **Upper Yangtze River** | **Mekong River** | **Salween River** |
| --- | --- | --- | --- | --- | --- |
|  | **n=15** | **n=8/7** | **n=7** | **n=12/11** | **n=3** |
| Dorsal fin | 8 (15) | 8 (8) | 8 (6), 9(1) | 8 (12) | 8 (3) |
| Pectoral fin | 14 (2), 15 (4), 16 (7), 17 (2) | 15 (5), 16 (3) | 15 (2), 16 (2), 17 (3) | 14 (2), 15 (6), 16 (2), 17 (2) | 14 (1), 15 (2) |
| Pelvic fin | 9 (11), 10 (4) | 9 (5), 10(3) | 9 (7) | 8(1), 9 (11) | 9 (3) |
| Anal fin | 5 (15) | 5 (8) | 5 (7) | 5 (12) | 5 (3) |
| Lateral line scale | 53 (1), 54 (1), 55 (3), 56 (3), 57 (2), 58 (5) | 51 (2),52(3), 53(2), 54 (1) | 52 (2), 53 (2), 54 (3) | 54 (1), 55 (4), 56 (4), 57 (1), 58 (2) | 53 (2), 55 (1) |
| Scale row above lateral line | 9.5 (3), 10 (1), 10.5 (8), 11 (3) | 9 (1), 9.5(4), 10(1), 10.5 (1), 11 (1) | 9 (1), 9.5 (1), 10 (1), 10.5 (2), 11.5 (2) | 9.5 (1), 10.5 (4), 11.5 (4), 12 (3) | 10.5 (2), 11.5 (1) |
| Scale row below lateral line | 5.5 (3), 6 (3), 6.5 (9) | 5.5(3),6.5 (4),7.5(1) | 5.5 (1), 6 (1), 6.5 (3), 7.5 (1), 8.5 (1) | 5.5 (2), 6.5 (9), 7.5 (1) | 5.5 (2), 6 (1) |
| First pterygiophore position | 15 (2), 16 (11), 17(2) | 16 (3), 17 (4) | 16 (4), 17 (3) | 17 (11) | 18 (3) |
| Second pterygiophore position | 17 (7), 18 (8) | 18 (3),19(4) | 18 (3), 19 (4) | 19 (8), 20 (3) | 20 (2), 21 (1) |
| Trunk vertebrae | 30 (2), 31 (8), 32 (5) | 31 (5), 32 (2) | 31 (1), 32 (4), 33 (2) | 31 (5), 32 (4), 33 (2) | 31 (1), 32 (2) |
| Vertebral column | 48(1),49 (4), 50 (10) | 49 (6), 50 (1) | 50 (6),51(1) | 48 (1), 49 (3), 50 (7) | 50 (2), 52 (1) |
