# Supplementary material for: Molecular Phylogeny and Biogeography of Percocypris (Cyprinidae, Teleostei)
Source: PLoS One. 2013 Jun 4;8(6):e61827. doi: 10.1371/journal.pone.0061827 (PMC3672144; doi:10.1371/journal.pone.0061827)
Supplement: Table S5 — Primary BPA matrix listing the distribution of Percocypris , along with the binary codes representing the phylogenetic relationships among the genus. (DOC) [file pone.0061827.s007.doc]

Table S5. Primary BPA matrix listing the distribution of *Percocypris*, along with the binary codes representing the phylogenetic relationships among the genus.

| Area | Species | 1 | 2 | 3 | 4 | 5 | 6 | 7 | 8 | 9 |
| --- | --- | --- | --- | --- | --- | --- | --- | --- | --- | --- |
| A | *P. pingi* | 1 | 0 | 0 | 0 | 0 | 0 | 1 | 0 | 1 |
| B | *P.* sp1 | 0 | 1 | 0 | 0 | 0 | 1 | 1 | 0 | 1 |
| C | *P. regani* | 0 | 0 | 1 | 0 | 0 | 1 | 1 | 0 | 1 |
| D | *P. retrodorslis* | 0 | 0 | 0 | 1 | 1 | 0 | 0 | 1 | 1 |
| E | *P.* sp2 | 0 | 0 | 0 | 0 | 0 | 0 | 0 | 1 | 1 |

A - Upper Yangtze River, B - Upper Pearl River, C - Fuxian Lake, D - Mekong River, E - Salween River.
